# Evaluation of the Protective Effect of a Well-Characterized n-Hexane Extract of Black Soldier Fly Larvae (*Hermetia illucens*) Against Acrylamide-Induced Testicular Damage in Male Rats: A Histopathological, Oxidative Stress, Inflammatory, and Molecular Study

**DOI:** 10.5812/ijpr-173454

**Published:** 2026-08-22

**Authors:** Fateme Heidari, Tahereh Zarei Taher, Yalda Arast

**Affiliations:** 1 Department of Anatomical Sciences, School of Medicine, Qom University of Medical Sciences Qom Iran; 2 Department of Tissue Engineering and Applied Cell Sciences, Faculty of Medicine, Qom University of Medical Sciences Qom Iran; 3 Student Research Committee, Qom University of Medical Sciences Qom Iran; 4 Research Center for Environmental Pollutants, Qom University of Medical Sciences Qom Iran

**Keywords:** Acrylamide, *Hermetia illucens*, Testicular Toxicity, Oxidative Stress, Inflammation, Apoptosis, Nrf2, Lauric Acid, Circular Economy

## Abstract

**Background:**

Acrylamide (ACR) is a ubiquitous contaminant present in environmental and dietary sources and is formed particularly in starchy foods during high-temperature processing. Prolonged exposure to ACR is associated with testicular dysfunction mediated by oxidative injury, inflammatory responses, and apoptotic cell death. Black soldier fly larvae (BSFL; *Hermetia illucens*) represent a sustainable source of bioactive lipids, notably lauric acid, which has recognized antioxidant and anti-inflammatory properties. Importantly, BSFL can be cultivated on organic waste, converting low-value materials into lipid-enriched biomass with therapeutic potential, consistent with the principles of the circular economy.

**Objectives:**

This study was designed, for the first time, to investigate the protective potential of a chemically characterized n-hexane extract of BSFL against acrylamide-induced testicular toxicity in a rat model.

**Methods:**

Thirty-five adult male Wistar rats were allocated to five experimental groups (n = 7 per group): vehicle control, ACR (20 mg/kg), ACR plus BSFL180 (180 mg/kg), ACR plus BSFL360 (360 mg/kg), and BSFL360 alone. All treatments were administered orally for 28 consecutive days. Serum levels of follicle-stimulating hormone (FSH), luteinizing hormone (LH), and dihydrotestosterone (DHT) were measured. Testicular specimens were analyzed for oxidative stress markers (malondialdehyde [MDA], superoxide dismutase [SOD], catalase [CAT], glutathione peroxidase [GPx], reduced glutathione [GSH]), inflammatory cytokines (TNF-α, IL-6, IL-1β), and apoptosis-related proteins (Nrf2, Bcl-2, Bax). Histological assessment was performed using H&E staining.

**Results:**

ACR administration markedly reduced serum FSH, LH, and DHT levels; increased MDA and pro-inflammatory cytokine levels; suppressed antioxidant enzyme activities and GSH content; and induced severe histopathological damage. Co-administration of BSFL extract, particularly at 360 mg/kg, dose-dependently counteracted these detrimental effects. Western blot analysis showed that BSFL extract upregulated Nrf2 and Bcl-2 expression while downregulating Bax, thereby restoring the Bax/Bcl-2 balance. The extract alone produced no adverse effects.

**Conclusions:**

The n-hexane extract of BSFL exerts substantial protective effects against ACR-induced testicular toxicity by restoring hormonal balance, mitigating oxidative stress, suppressing inflammation, and modulating Nrf2 and Bax/Bcl-2 signaling. These findings highlight the therapeutic potential of BSFL as a sustainable, cost-effective natural product within a circular bioeconomy framework and a novel approach to preventing chemically induced testicular injury.

## 1. Background

Acrylamide is a major environmental and occupational contaminant that is widely present in industrial settings, including paper manufacturing, wastewater treatment, and cosmetic production, as well as in laboratory research applications (1, 2). In addition to industrial sources, acrylamide is thermally generated in carbohydrate-rich foods during common cooking practices, including frying, baking, and roasting. This process primarily occurs via the Maillard reaction between the amino acid asparagine and reducing sugars (3, 4). Accordingly, acrylamide is routinely detected in commonly consumed foods such as potato chips, crackers, bread, and coffee, making long-term, low-dose exposure largely unavoidable in human populations (5).

Extensive experimental evidence accumulated over recent decades has classified acrylamide as a potent reproductive toxicant. In animal models, repeated administration at doses of 5 - 50 mg/kg body weight causes marked testicular injury, characterized by reduced testosterone secretion, loss of Leydig cells, germ cell degeneration, the presence of multinucleated giant cells, and severe disruption of spermatogenesis (6-9). The underlying pathophysiological mechanisms are complex, with oxidative damage, inflammatory responses, and programmed cell death recognized as key contributors to ACR-induced testicular toxicity (10). Notably, ACR exposure induces the overproduction of reactive oxygen species (ROS), depletion of intracellular antioxidant defenses, increased lipid peroxidation reflected by elevated malondialdehyde (MDA), and upregulated expression of inflammatory mediators, including tumor necrosis factor-alpha (TNF-α), interleukin-1 beta (IL-1β), and interleukin-6 (IL-6) (11). Although plant-derived flavonoids and other phytochemicals have shown promise as protective agents against ACR, their practical application is often limited by low bioavailability, high manufacturing costs, and concerns regarding environmental sustainability. Therefore, identifying alternative, eco-friendly, and economically viable sources of bioactive compounds with comparable or improved efficacy remains a research priority.

The use of insects and insect-derived products in traditional healing practices has a long history across diverse cultures, particularly for managing inflammatory and reproductive conditions. More recently, insect larvae have gained attention as a renewable source of bioactive lipids, providing a sustainable alternative to conventional plant-based remedies. Among these, black soldier fly larvae (BSFL; *Hermetia illucens*) have emerged as a particularly promising candidate because of their high content of lipids, proteins, and bioactive molecules, including medium-chain fatty acids (especially lauric acid), antimicrobial peptides, and antioxidants (12).

Previous analytical studies have shown that the n-hexane fraction of BSFL is highly enriched in lauric acid (13, 14), a medium-chain fatty acid with recognized antioxidant and anti-inflammatory potential (15-17). Lauric acid has been reported to enhance antioxidant enzyme activity, reduce lipid peroxidation, and preserve testicular tissue architecture in experimental models of reproductive injury (17). However, the protective effects of BSFL n-hexane extract against ACR-induced testicular injury have not been previously investigated.

## 2. Objectives

Accordingly, the present study was conducted to determine whether BSFL n-hexane extract can alleviate ACR-mediated testicular damage by reducing oxidative stress and inhibiting inflammatory pathways. Protective efficacy was evaluated using comprehensive histological analysis, quantification of inflammatory markers, and assessment of apoptosis-related proteins (Nrf2, Bcl-2, and Bax) in testicular tissue.

## 3. Methods

### 3.1. Chemicals and Reagents

Acrylamide (purity ≥ 99%) was obtained from Sigma-Aldrich (St. Louis, MO, USA). The n-hexane extract of BSFL was prepared according to the procedure described in Section 3.2. Corn oil, used as the vehicle, and all other analytical-grade reagents were obtained from domestic suppliers. Commercial ELISA kits were used to measure hormonal parameters (luteinizing hormone [LH], follicle-stimulating hormone [FSH], dihydrotestosterone [DHT]), oxidative stress indicators (superoxide dismutase [SOD], catalase [CAT], glutathione peroxidase [GPx], reduced glutathione [GSH], malondialdehyde [MDA]), and inflammatory markers (interleukin-1β [IL-1β], interleukin-6 [IL-6], tumor necrosis factor-alpha [TNF-α]). All kits were obtained from reliable vendors, including Sunred (Shanghai, China) or equivalent certified providers.

### 3.2. Preparation of Black Soldier Fly Larvae n-Hexane Extract

The n-hexane extract of BSFL (*Hermetia illucens*) used in this study was developed as a technological formulation at the Growth Center of Qom University of Medical Sciences, Qom, Iran. Larvae were reared on a diet consisting of fruit residues and olive pomace under controlled environmental conditions (27 ± 2 °C, 65 - 70% relative humidity). At the prepupal stage (approximately 14 - 16 days post-hatching), larvae were collected, washed with distilled water, and dehydrated in an oven at 60 °C for 24 hours. The dried material was then pulverized to a fine powder using a mechanical grinder and stored at −20 °C until extraction.

Lipid extraction was performed by subjecting the powdered biomass to Soxhlet extraction with n-hexane for approximately 3 hours, using a solvent-to-sample ratio of 10:1 (v/w). The extraction was conducted with continuous reflux (one extraction cycle). The solvent was evaporated under reduced pressure using a rotary evaporator at 40 °C (12, 14). The extraction yield was approximately 22% (w/w) based on dry larval weight, consistent with typical ranges reported for n-hexane extraction of black soldier fly larvae oil (12, 14). The resulting crude oil was transferred to amber vials, flushed with nitrogen to prevent oxidative degradation, and stored at −20 °C.

### 3.3. Fatty Acid Profiling of BSFL Extract Using GC-MS

The fatty acid composition of the BSFL n-hexane extract was determined by gas chromatography-mass spectrometry (GC-MS). Analyses were performed at the Behesht-e-Aein Knowledge-Based Laboratory, accredited by the Iran Food and Drug Administration (IFDA). Fatty acid methyl esters (FAMEs) were identified by comparison with the NIST spectral library and authenticated FAME standards (Supelco 37-component FAME mix, Sigma-Aldrich, USA). The relative abundance of each fatty acid was calculated using peak area normalization. The complete fatty acid profile is presented in Table 1.

**Table 1.** Fatty Acid Composition of BSFL n-Hexane Oil Extract (GC-MS Analysis) ^a^

Fatty Acid	Percentage
**Lauric acid (C12:0)**	48.00
**Palmitic acid (C16:0)**	16.00
**Oleic acid (C18:1)**	12.00
**Myristic acid (C14:0)**	9.90
**Linoleic acid (C18:2)**	3.80
**Other fatty acids**	10.30
**Total**	100.00

^a^ Values represent mean of triplicate analyses.

### 3.4. Animals

Thirty-five adult male Wistar rats (*Rattus norvegicus*), weighing 200 - 250 g (approximately 10 - 12 weeks old), were obtained from the institutional breeding facility. Animals were housed in polypropylene cages under standardized environmental conditions: temperature (22 ± 2 °C), relative humidity (50 - 60%), and a 12-hour light/dark cycle. They had free access to a standard pellet diet and drinking water throughout the acclimatization and experimental periods. All experimental procedures followed relevant guidelines for laboratory animal care and were approved by the Institutional Animal Ethics Committee of Qom University of Medical Sciences (Code: IR.MUQ.AEC.1404.020).

### 3.5. Experimental Design

After a one-week acclimatization period, rats were randomly assigned to five experimental groups, each comprising seven animals (n = 7), in accordance with previous studies to ensure adequate statistical power while adhering to the 3Rs principle (Replacement, Reduction, Refinement):

- Group 1 (Control): Received corn oil (2 mL/kg) daily by oral gavage for 28 days.

- Group 2 (ACR): Received acrylamide (20 mg/kg) dissolved in corn oil (2 mL/kg) daily for 28 days, based on prior research demonstrating reproducible testicular toxicity without mortality (6, 18).

- Group 3 (ACR + BSFL180): Received acrylamide (20 mg/kg) plus BSFL extract (180 mg/kg) in corn oil (2 mL/kg) for 28 days.

- Group 4 (ACR + BSFL360): Received acrylamide (20 mg/kg) plus BSFL extract (360 mg/kg) in corn oil (2 mL/kg) for 28 days.

- Group 5 (BSFL360 alone): Received BSFL extract (360 mg/kg) in corn oil (2 mL/kg) alone for 28 days.

The BSFL extract doses (180 and 360 mg/kg) were selected based on its lipid profile, particularly the 48% lauric acid content identified by GC-MS analysis (Table 1). These doses correspond to daily lauric acid intakes of approximately 86 and 173 mg/kg, which are within the established effective range (100 - 250 mg/kg) for lauric acid in toxin-challenged rodent models (19). These doses are also consistent with our previous toxicological work using BSFL extract under similar conditions (20). The higher dose was included to evaluate dose-dependent effects and confirm efficacy. The ACR dose (20 mg/kg) was selected based on previous studies demonstrating reproducible testicular toxicity without mortality (6, 18). The 28-day treatment period was selected according to established testicular toxicity models and to allow sufficient time for the development of oxidative stress and inflammatory responses (6, 18). All treatments were administered daily between 9:00 and 10:00 AM to minimize circadian variability. Body weight was recorded at baseline and then weekly, with a final measurement on day 29 before euthanasia.

### 3.6. Sample Collection

Approximately 24 hours after the final treatment (day 29), animals were weighed and deeply anesthetized by intraperitoneal injection of ketamine (50 mg/kg) and xylazine (10 mg/kg) (21). Adequate anesthesia was confirmed by the absence of a pedal withdrawal reflex. Blood samples were collected by cardiac puncture using sterile syringes and transferred to plain tubes for serum separation. After clotting at room temperature for 30 minutes, samples were centrifuged at 3000 rpm for 15 minutes at 4 °C. Serum was then aliquoted and stored at −20 °C for subsequent hormonal and biochemical analyses.

After blood collection, rats were euthanized by cervical dislocation under deep anesthesia. Both testes were rapidly excised, cleared of adhering fat and connective tissue, and weighed. The right testis was snap-frozen in liquid nitrogen and stored at −80 °C for biochemical and molecular evaluations, whereas the left testis was fixed in 10% neutral buffered formalin for histopathological examination.

### 3.7. Hormonal Assays

Serum FSH, LH, and DHT levels were quantified using commercial ELISA kits (Teb Pazhouhan Razi, Tehran, Iran) according to the manufacturer’s instructions (22). Absorbance was measured using a microplate reader at the appropriate wavelengths. Hormone concentrations were calculated from standard curves and reported as mIU/mL (FSH, LH) or pg/mL (DHT).

### 3.8. Assessment of Oxidative Stress Parameters in Testicular Tissue

For oxidative stress assessment, frozen testicular specimens were homogenized in ice-cold phosphate buffer (0.1 M, pH 7.4). After centrifugation at 10,000 × g for 15 minutes at 4 °C, the supernatant was collected. Protein content was determined using the Bradford method with bovine serum albumin (BSA) as the standard (22). The following oxidative stress parameters were measured using commercial kits (Navand Lab, Tehran, Iran) (23-25):

Superoxide Dismutase (SOD) Activity: Assessed by inhibition of nitroblue tetrazolium (NBT) reduction; results expressed as U/mg protein (23, 24).

Catalase (CAT) Activity: Assessed by monitoring hydrogen peroxide (H_2_O_2_) decomposition at 240 nm; results expressed as U/mg protein (23, 25).

Glutathione Peroxidase (GPx) Activity: Measured by NADPH oxidation in the presence of glutathione and H_2_O_2_; results expressed as U/mg protein (23, 24).

Reduced Glutathione (GSH) Level: Quantified using Ellman’s reagent (DTNB) at 412 nm; results expressed as μmol/g tissue (23, 25).

Malondialdehyde (MDA) Level: Determined by reaction with thiobarbituric acid (TBA) at 532 nm; results expressed as nmol/mg protein (23, 24).

### 3.9. Assessment of Inflammatory Markers in Testicular Tissue

Inflammatory cytokine levels in testicular homogenates were measured using ELISA kits (CN: KPG-TNFα-48, KPG-RIL1β, KPG-RIL6; Karmania Pars Gene, Tehran, Iran) and an IL-1β kit (IBL, code 27193) for TNF-α, IL-6, and IL-1β (25). Cytokine levels were normalized to total protein and expressed as pg/mg protein.

### 3.10. Assessment of Apoptosis-Regulatory Proteins by Western Blotting

Western blotting was used to assess Nrf2, Bcl-2, and Bax expression. Testicular tissue was lysed in RIPA buffer and centrifuged at 14,000 rpm for 20 minutes at 4 °C. Protein concentration was determined using the Bradford method (DNAbioTech, Iran). Samples containing 20 μg of protein were mixed with Laemmli buffer, boiled for 5 minutes, and separated by SDS-PAGE. Proteins were transferred to PVDF membranes (0.2 μm, Bio-Rad), blocked with 5% BSA in 0.1% Tween 20 for 1 hour, and incubated overnight at 4 °C with primary antibodies against Nrf2 (1:1000), Bcl-2 (1:1000), Bax (1:1000), and β-actin (1:2500). After washing with TBST, membranes were incubated with an HRP-conjugated secondary antibody (1:10000). Bands were visualized using ECL and quantified using ImageJ software (NIH, USA). Results were normalized to β-actin and expressed as fold change relative to the control group.

### 3.11. Histopathological Examination

Fixed testicular tissues were processed through a graded alcohol series, cleared in xylene, and embedded in paraffin. Sections (4 - 5 μm) were stained with hematoxylin and eosin (H&E) using standard protocols (18, 21). For morphometric analysis, 10 randomly selected seminiferous tubules from three non-consecutive sections per animal were examined under a light microscope by a pathologist blinded to group assignments. Germinal epithelial height was measured using ImageJ software (NIH, USA) and averaged per animal to ensure that the analysis represented the individual animal rather than individual microscopic fields (18, 21). Histological changes were scored on a 0 - 3 scale (0: normal, 1: mild, 2: moderate, 3: severe).

### 3.12. Statistical Analysis

Statistical analyses were performed using SPSS (version 22.0). Data are presented as mean ± standard deviation (SD). Normality was assessed using the Shapiro-Wilk test. Group comparisons were performed using one-way ANOVA followed by Tukey’s post hoc test for parametric data, which inherently controls for multiple pairwise comparisons by maintaining the family-wise error rate at α = 0.05, and the Kruskal-Wallis test followed by the Mann-Whitney U test for non-parametric data (e.g., histology scores). The statistical unit for all analyses was the individual animal (n = 7 per group). A P value < 0.05 was considered statistically significant.

### 3.13. Ethical Considerations

All experimental procedures strictly followed the NIH Guide for the Care and Use of Laboratory Animals. The study design received prior approval from the Institutional Animal Ethics Committee of Qom University of Medical Sciences (Code: IR.MUQ.AEC.1404.020). Efforts were made to minimize suffering and reduce animal numbers to the minimum required for statistically valid outcomes. Cervical dislocation under deep anesthesia was performed in accordance with AVMA guidelines (2020) and approved as a humane procedure.

## 4. Results

In the present study, we evaluated the protective effect of the n-hexane extract of black soldier fly larvae (BSFL) against acrylamide (ACR)-induced testicular toxicity in male Wistar rats. ACR administration (20 mg/kg) for 28 consecutive days induced significant testicular damage, as evidenced by hormonal imbalance, oxidative stress, inflammation, apoptosis, and histopathological alterations. Co-treatment with BSFL extract at two doses (180 and 360 mg/kg) ameliorated ACR-induced testicular injury in a dose-dependent manner. Administration of BSFL extract alone (360 mg/kg) did not cause adverse effects on the measured parameters, confirming its safety profile. The detailed results are presented in the following subsections.

### 4.1. Hormonal Assays

**Figure 1.**
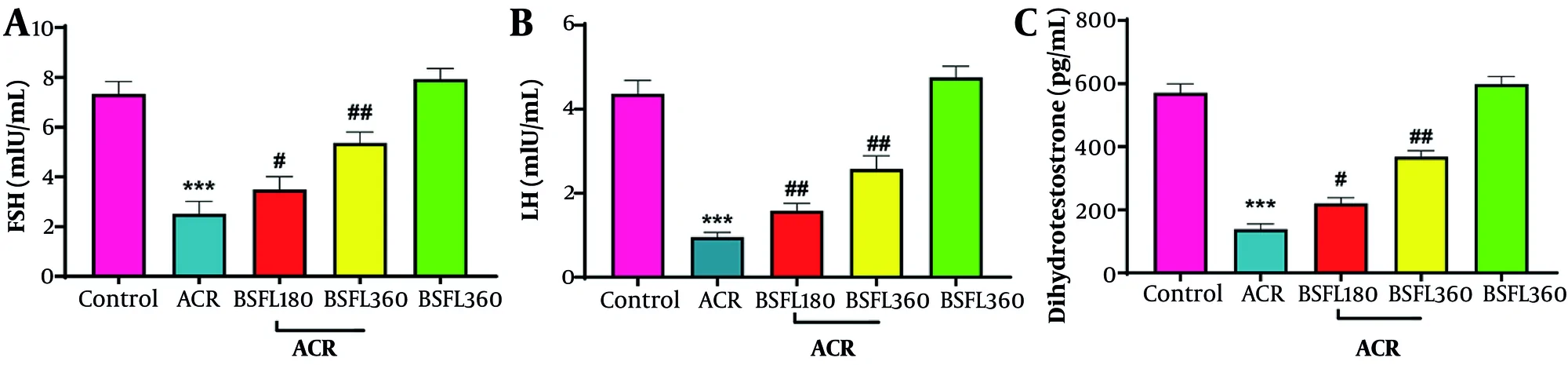
Effects of acrylamide (ACR) and black soldier fly larvae extract (BSFL) on serum levels of (A) follicle-stimulating hormone (FSH), (B) luteinizing hormone (LH), and (C) dihydrotestosterone (DHT). Data are expressed as mean ± SD (n = 7). ***P < 0.001 vs. control group. #P < 0.05, ##P < 0.01, ###P < 0.001 vs. ACR group (one-way ANOVA followed by Tukey's post hoc test). ACR: acrylamide (20 mg/kg); BSFL180: BSFL extract (180 mg/kg); BSFL360: BSFL extract (360 mg/kg); BSFL360 alone: BSFL extract (360 mg/kg) administered alone.

The effects of acrylamide (ACR) and black soldier fly larvae extract (BSFL) on serum levels of follicle-stimulating hormone (FSH), luteinizing hormone (LH), and dihydrotestosterone (DHT) are shown in Figure 1A-C.

FSH and LH Levels: ACR administration alone significantly decreased serum FSH and LH levels compared with the control group (P < 0.001). Co-treatment with BSFL extract at 180 mg/kg (ACR + BSFL180) resulted in a partial but significant increase in both FSH and LH levels compared with the ACR group (P < 0.05). The higher dose of BSFL extract (ACR + BSFL360) exerted a stronger ameliorative effect, significantly restoring FSH and LH levels toward normal values (P < 0.01). Administration of BSFL extract alone (BSFL360) did not adversely affect FSH or LH levels; rather, it maintained these hormones at levels comparable to or slightly higher than those in the control group, confirming the safety of the extract.

DHT Levels: Consistent with the effects on FSH and LH, DHT levels were markedly reduced following ACR exposure (P < 0.001). Co-treatment with BSFL180 partially restored DHT levels (P < 0.05), whereas BSFL360 showed a more pronounced protective effect, significantly increasing DHT levels compared with the ACR group (P < 0.01). The BSFL360 alone group exhibited DHT levels comparable to those in the control group, further supporting the nontoxic profile of the extract.

These findings indicate that ACR-induced suppression of the hypothalamic-pituitary-gonadal (HPG) axis was effectively counteracted by BSFL extract in a dose-dependent manner, with the higher dose (360 mg/kg) showing superior protective efficacy.

### 4.2. Oxidative Stress Parameters

**Figure 2.**
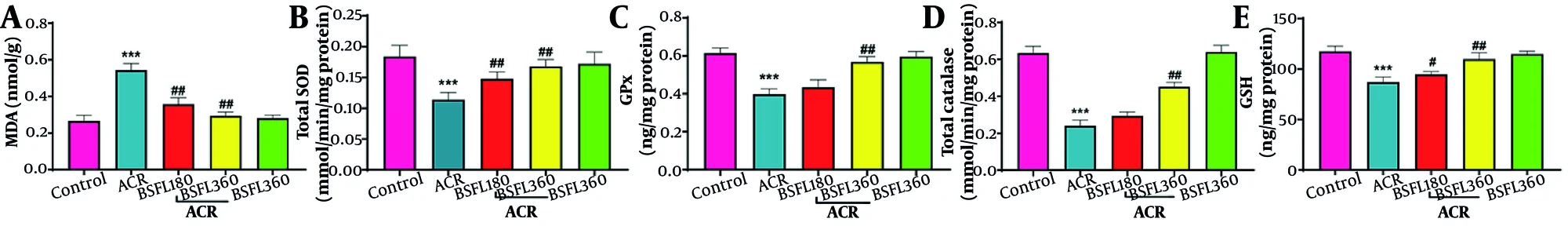
Effects of ACR and BSFL extract on testicular oxidative stress parameters: (A) malondialdehyde (MDA), (B) superoxide dismutase (SOD), (C) glutathione peroxidase (GPx), (D) catalase (CAT), and (E) reduced glutathione (GSH). Data are expressed as mean ± SD (n = 7). ***P < 0.001 vs. control group. #P < 0.05, ##P < 0.01, ###P < 0.001 vs. ACR group (one-way ANOVA followed by Tukey's post hoc test).

The effects of acrylamide (ACR) and black soldier fly larvae extract (BSFL) on testicular oxidative stress markers, including malondialdehyde (MDA), superoxide dismutase (SOD), glutathione peroxidase (GPx), reduced glutathione (GSH), and catalase (CAT), are shown in Figure 2A-E.

### 4.2.1. Lipid Peroxidation (MDA Level):

As shown in Figure 2A, ACR administration alone significantly increased testicular MDA levels compared with the control group, indicating enhanced lipid peroxidation (P < 0.001). Co-treatment with BSFL180 partially reduced MDA levels (P < 0.05 vs. ACR group). The higher dose of BSFL extract (ACR + BSFL360) exerted a more pronounced protective effect, significantly lowering MDA levels compared with the ACR group (P < 0.01). Administration of BSFL360 alone maintained MDA levels comparable to those in the control group, confirming the nontoxic nature of the extract.

### 4.2.2. Antioxidant Enzyme Activities (SOD, GPx, and CAT):

ACR exposure significantly decreased testicular SOD, GPx, and CAT activities compared with the control group (P < 0.001), indicating compromised antioxidant defenses (Figure 2B, 2C, and 2D). Co-treatment with BSFL180 partially restored SOD, GPx, and CAT activities (P < 0.05 vs. ACR group). The higher dose (ACR + BSFL360) produced a stronger ameliorative effect, significantly increasing SOD, GPx, and CAT activities compared with the ACR group (P < 0.01). The BSFL360 alone group showed SOD, GPx, and CAT activities similar to those in the control group.

### 4.2.3. Reduced Glutathione (GSH Level):

As shown in Figure 2E, ACR administration markedly depleted testicular GSH levels compared with the control group (P < 0.001). Co-treatment with BSFL180 partially restored GSH levels (P < 0.05 vs. ACR group), whereas ACR + BSFL360 significantly increased GSH levels (P < 0.01 vs. ACR group). The BSFL360 alone group maintained GSH levels comparable to those in the control group.

These findings indicate that BSFL extract effectively ameliorated ACR-induced oxidative stress in testicular tissue by reducing lipid peroxidation and restoring antioxidant enzyme activities and glutathione levels in a dose-dependent manner.

### 4.3. Inflammatory Markers

**Figure 3.**
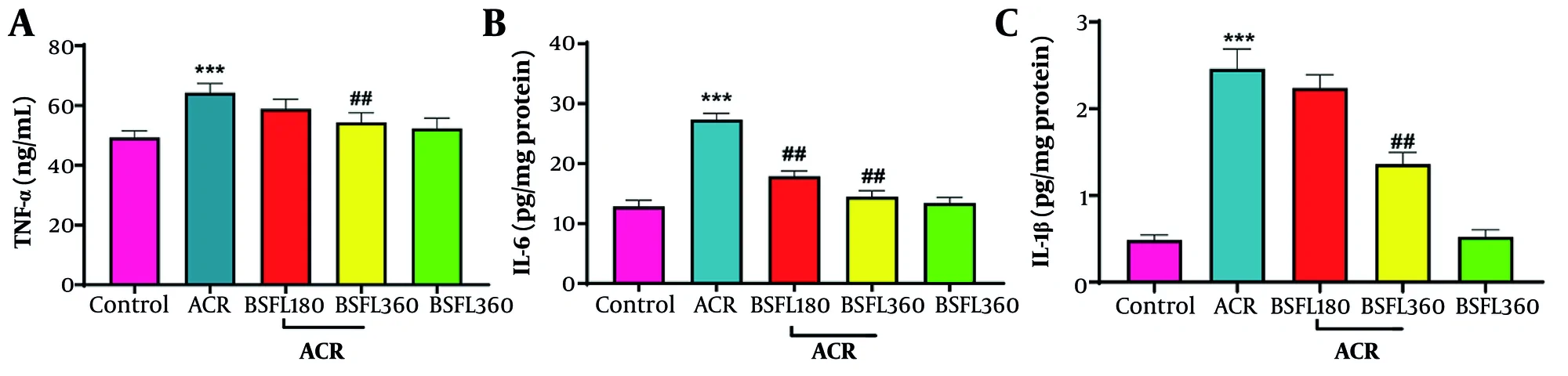
Effects of ACR and BSFL extract on testicular inflammatory markers: (A) tumor necrosis factor-alpha (TNF-α), (B) interleukin-6 (IL-6), and (C) interleukin-1 beta (IL-1β). Data are expressed as mean ± SD (n = 7). ***P < 0.001 vs. control group; #P < 0.05, ##P < 0.01 vs. ACR group (one-way ANOVA followed by Tukey's post hoc test).

The effects of ACR and BSFL extract on testicular levels of pro-inflammatory cytokines, including tumor necrosis factor-alpha (TNF-α), interleukin-6 (IL-6), and interleukin-1 beta (IL-1β), are shown in Figure 3A-C.

### 4.3.1. TNF-Α Level:

As shown in Figure 3A, ACR administration alone significantly increased the testicular TNF-α level compared with the control group (P < 0.001). Co-treatment with BSFL180 partially reduced the TNF-α level (P < 0.05 vs. ACR group). The higher dose of BSFL extract (ACR + BSFL360) exerted a more pronounced anti-inflammatory effect, significantly lowering the TNF-α level compared with the ACR group (P < 0.01). Administration of BSFL360 alone maintained the TNF-α level comparable to that in the control group.

### 4.3.2. IL-6 Level:

As shown in Figure 3B, ACR exposure significantly increased the testicular IL-6 level compared with the control group (P < 0.001). Co-treatment with BSFL180 partially attenuated this increase (P < 0.05 vs. ACR group). The higher dose (ACR + BSFL360) demonstrated a stronger protective effect, significantly reducing the IL-6 level compared with the ACR group (P < 0.01). The BSFL360 alone group showed an IL-6 level similar to that in the control group.

### 4.3.3. IL-1β Level:

Similar to TNF-α and IL-6, ACR administration markedly increased the testicular IL-1β level compared with the control group (P < 0.001) (Figure 3C). Co-treatment with BSFL180 partially reduced the IL-1β level (P < 0.05 vs. ACR group), whereas ACR + BSFL360 significantly decreased the IL-1β level (P < 0.01 vs. ACR group). The BSFL360 alone group maintained the IL-1β level comparable to that in the control group.

These findings indicate that BSFL extract effectively suppressed ACR-induced testicular inflammation by reducing the levels of the pro-inflammatory cytokines TNF-α, IL-6, and IL-1β in a dose-dependent manner.

### 4.4. Histopathological Examination

**Figure 4.**
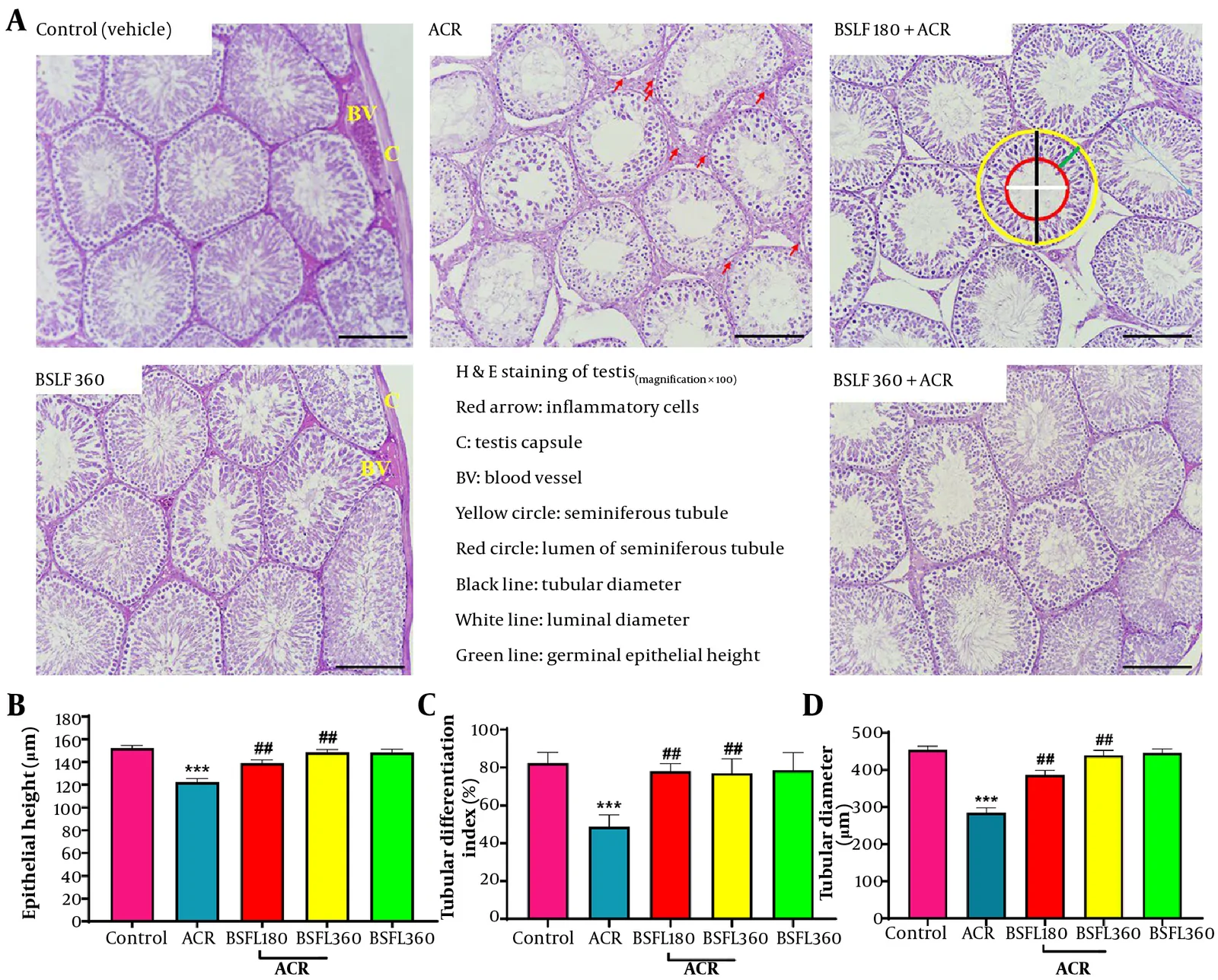
(A) Representative photomicrographs of hematoxylin and eosin (H&E)-stained testicular sections (magnification × 100). Control: normal testicular architecture; ACR: severe degeneration, inflammatory cells (red arrows), and reduced luminal sperm; ACR + BSFL180: moderate improvement; ACR + BSFL360: significant restoration of testicular structure; BSFL360 alone: normal architecture comparable to control. C: testis capsule, BV: blood vessel. (B) Quantitative analysis of germinal epithelial height. (C) Quantitative analysis of inflammatory cell infiltration (%). (D) Quantitative analysis of germinal epithelial degeneration (%). Data are expressed as mean ± SD (n = 7). ***P < 0.001 vs. control group; #P < 0.05, ##P < 0.01 vs. ACR group (one-way ANOVA followed by Tukey's post hoc test).

The protective effect of BSFL extract against ACR-induced testicular damage was further evaluated by hematoxylin and eosin (H&E) staining. Representative photomicrographs of testicular sections from each experimental group are shown in Figure 4.

The control group showed normal testicular histoarchitecture, with well-organized seminiferous tubules, an intact germinal epithelium, and abundant spermatozoa within the lumina. The interstitial space contained normal Leydig cells, and no inflammatory cell infiltration was observed.

The ACR group exhibited severe testicular damage, characterized by disorganized seminiferous tubules, degeneration and thinning of the germinal epithelium, and a marked reduction of spermatozoa in the lumina. Inflammatory cell infiltration was clearly observed (indicated by red arrows), along with interstitial edema and vacuolization.

The ACR + BSFL180 group showed moderate improvement compared with the ACR group. The germinal epithelial height was partially restored, and the luminal sperm content was increased. However, mild inflammatory cell infiltration and slight epithelial disorganization were still evident.

The ACR + BSFL360 group demonstrated marked protection of testicular architecture. The seminiferous tubules showed near-normal organization, the germinal epithelial height was substantially restored, and the lumina contained abundant spermatozoa. Inflammatory cell infiltration was minimal.

The BSFL360 alone group exhibited normal testicular histoarchitecture comparable to that of the control group, confirming the nontoxic profile of the extract.

### 4.4.1. Morphometric Analysis:

Quantitative analysis of germinal epithelial height (Figure 4B) revealed a significant decrease in the ACR group compared with the control group (P < 0.001). Co-treatment with BSFL180 partially restored epithelial height (P < 0.05 vs. ACR group), whereas ACR + BSFL360 significantly increased epithelial height (P < 0.01 vs. ACR group). The BSFL360 alone group showed epithelial height comparable to that of the control group.

These histological and morphometric findings further support the protective effect of BSFL extract against ACR-induced testicular injury in a dose-dependent manner.

### 4.5. Western Blot Analysis of Apoptosis-Regulatory Proteins (Nrf2, Bcl-2, and Bax)

**Figure 5.**
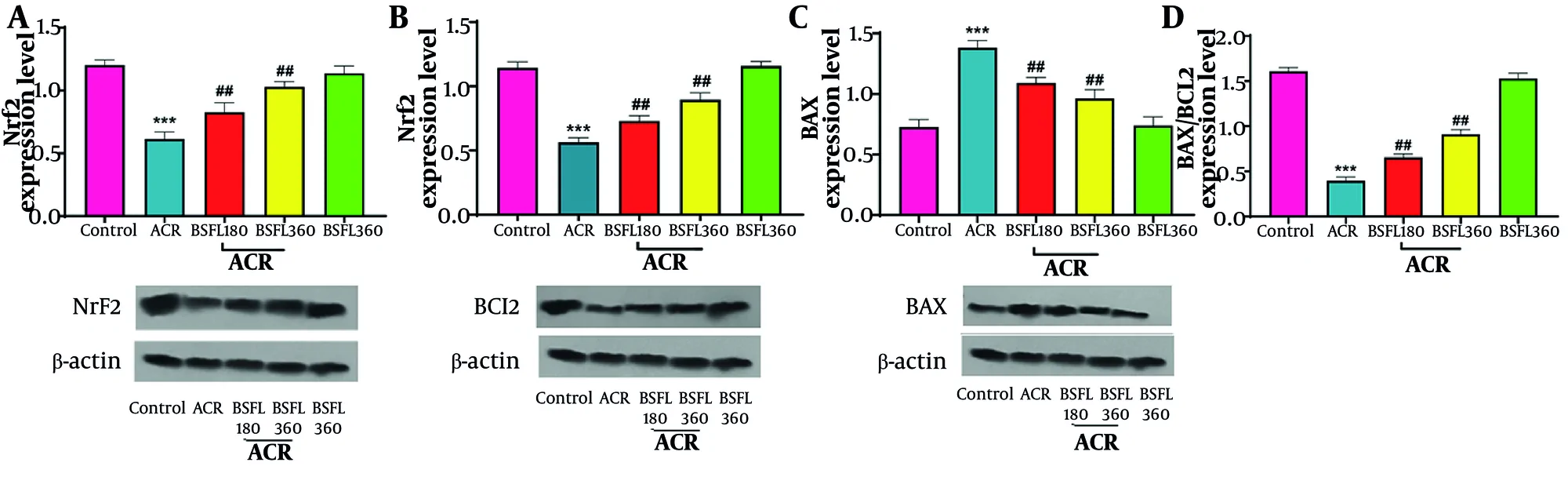
Effects of ACR and BSFL extract on protein expression levels of (A) Nrf2, (B) Bcl-2, (C) Bax, and (D) Bax/Bcl-2 ratio in testicular tissue, as determined by Western blot analysis. β-actin was used as an internal loading control. Data are expressed as mean ± SD (n = 3). ***P < 0.001 vs. control group; #P < 0.05, ##P < 0.01 vs. ACR group (one-way ANOVA followed by Tukey's post hoc test).

To further elucidate the molecular mechanisms underlying the protective effect of BSFL extract against ACR-induced testicular toxicity, the protein expression levels of nuclear factor erythroid 2-related factor 2 (Nrf2), B-cell lymphoma 2 (Bcl-2), and Bcl-2-associated X protein (Bax) were evaluated by Western blotting. The results are shown in Figure 5A-D.

### 4.5.1. Nrf2 Expression:

As shown in Figure 5A, ACR administration alone significantly downregulated testicular Nrf2 protein expression compared with the control group (P < 0.001). Co-treatment with BSFL extract at 180 mg/kg (ACR + BSFL180) partially restored Nrf2 expression (P < 0.05 vs. ACR group). The higher dose of BSFL extract (ACR + BSFL360) exerted a more pronounced effect, significantly upregulating Nrf2 expression compared with the ACR group (P < 0.01). Administration of BSFL360 alone maintained Nrf2 expression at levels comparable to those in the control group (P > 0.05 vs. control).

### 4.5.2. Bcl-2 Expression:

As shown in Figure 5B, ACR exposure significantly decreased testicular Bcl-2 protein expression compared with the control group (P < 0.001). Co-treatment with BSFL180 partially restored Bcl-2 expression (P < 0.05 vs. ACR group). The higher dose (ACR + BSFL360) significantly increased Bcl-2 expression compared with the ACR group (P < 0.01), restoring it to near-normal levels. The BSFL360 alone group showed Bcl-2 expression comparable to that of the control group.

### 4.5.3. Bax Expression:

As shown in Figure 5C, ACR administration significantly upregulated testicular Bax protein expression compared with the control group (P < 0.001). Co-treatment with BSFL180 partially reduced Bax expression (P < 0.05 vs. ACR group). The higher dose (ACR + BSFL360) exerted a stronger protective effect, significantly decreasing Bax expression compared with the ACR group (P < 0.01). The BSFL360 alone group maintained Bax expression at levels similar to those in the control group.

### 4.5.4. Bax/Bcl-2 Ratio:

The Bax/Bcl-2 ratio, which determines susceptibility to apoptosis, was markedly increased in the ACR group compared with the control group (P < 0.001) (Figure 5D). Co-treatment with BSFL180 partially reduced this ratio (P < 0.05 vs. ACR group), whereas ACR + BSFL360 significantly normalized the Bax/Bcl-2 ratio (P < 0.01 vs. ACR group). The BSFL360 alone group exhibited a Bax/Bcl-2 ratio comparable to that of the control group.

These findings suggest that BSFL extract exerts anti-apoptotic and antioxidant effects against ACR-induced testicular toxicity, at least in part, through upregulation of Nrf2 and modulation of the Bax/Bcl-2 signaling pathway.

## 5. Discussion

The present study provides, for the first time, a comprehensive evaluation of the protective effects of a chemically characterized n-hexane extract from black soldier fly larvae (BSFL) against acrylamide (ACR)-induced testicular toxicity in a rat model. Our results demonstrate that BSFL extract, at doses of 180 and 360 mg/kg, effectively counteracts ACR-induced hormonal imbalances, oxidative damage, inflammatory responses, alterations in apoptotic proteins, and structural damage in testicular tissue. The protective effects were dose dependent, with the 360 mg/kg dose showing greater efficacy. Importantly, BSFL extract administered alone did not produce any detectable adverse effects, supporting its safety profile.

ACR is a recognized reproductive toxicant that acts through multiple interconnected mechanisms, including oxidative injury, inflammation, and apoptosis (10, 11). In the present study, ACR exposure (20 mg/kg, 28 days) resulted in a notable disruption of circulating hormone levels, characterized by marked declines in FSH, LH, and DHT. This finding is consistent with previous reports that ACR interferes with the hypothalamic-pituitary-gonadal (HPG) axis, leading to reduced gonadotropin secretion and subsequent testicular dysfunction (6, 7, 21). The reduction in DHT, a critical androgen for normal spermatogenesis, further supports the detrimental effects of ACR on testicular steroidogenesis (17). The ability of BSFL extract to restore these hormone levels in a dose-dependent manner suggests a potential modulatory effect on the HPG axis, possibly mediated by its antioxidant and anti-inflammatory properties at the level of the hypothalamus and pituitary.

Oxidative stress is a central mechanism in ACR-mediated testicular damage (10, 11). ACR undergoes metabolic conversion to glycidamide, which promotes ROS generation and depletes endogenous antioxidant reserves. In our experimental setting, ACR exposure significantly increased testicular MDA, a well-established marker of lipid peroxidation, while simultaneously reducing the activities of SOD, CAT, GPx, and GSH. These findings are consistent with previous reports of ACR-induced oxidative stress in testicular tissue (7, 22, 26). The pronounced antioxidant activity of BSFL extract may be attributable, at least in part, to its high lauric acid content (48% of total fatty acids), which is comparable to that of coconut oil but derived from a more sustainable source. Lauric acid is a medium-chain fatty acid known to stimulate endogenous antioxidant enzymes and inhibit lipid peroxidation (15, 17). However, because this study evaluated a complex extract rather than purified lauric acid, other bioactive constituents (including other medium-chain fatty acids, tocopherols, and antimicrobial peptides) may also contribute to the overall protective effect (12, 14). A direct comparison with purified lauric acid in future studies would be valuable to determine whether the protective activity is primarily attributable to lauric acid or to the combined lipid profile of the extract (15, 17). Moreover, other BSFL components, including tocopherols and antimicrobial peptides, may synergistically enhance the overall antioxidant effect (12, 14).

Inflammation also plays a pivotal role in ACR-induced testicular toxicity (10). ACR is known to activate the NF-κB pathway, resulting in increased production of pro-inflammatory cytokines such as TNF-α, IL-1β, and IL-6 (11, 23). Our findings showed that ACR markedly upregulated these inflammatory mediators, whereas BSFL extract significantly suppressed them in a dose-dependent manner. This anti-inflammatory activity is likely mediated by inhibition of NF-κB activation, consistent with previous reports on lauric acid and related lipids (16, 17). By inhibiting NF-κB signaling, BSFL extract may reduce the transcription of inflammatory genes, thereby attenuating ACR-induced testicular inflammation.

BSFL represents not only a source of bioactive molecules but also a sustainable tool for waste management. In contrast to plant-derived extracts that require land, water, and agricultural inputs, BSFL can be cultivated on organic waste, converting low-value refuse into lipid-rich biomass with therapeutic potential. This circular economy framework promotes environmental sustainability and provides a scalable, economically viable approach for producing natural bioactive compounds, positioning BSFL as an important component of the bioeconomy.

The regulation of apoptotic proteins is critical for maintaining testicular homeostasis and spermatogenesis (10). In our study, ACR decreased Bcl-2 and increased Bax, resulting in an elevated Bax/Bcl-2 ratio indicative of mitochondria-dependent apoptosis. This finding is consistent with evidence that ACR induces apoptosis via the mitochondrial pathway (7, 24). BSFL extract reversed these changes by restoring Bcl-2 and reducing Bax, thereby normalizing the ratio. Mechanistically, this effect may be linked to Nrf2 upregulation, a master regulator of more than 200 cytoprotective genes, including antioxidant enzymes and anti-apoptotic proteins such as Bcl-2. The observed increase in Nrf2 protein expression with BSFL extract suggests possible involvement of the Nrf2/antioxidant response element (ARE) pathway in enhancing cellular antioxidant defense and shifting the Bax/Bcl-2 balance toward cell survival (15, 17). However, our data demonstrate changes in Nrf2 protein expression rather than direct evidence of Nrf2 nuclear translocation or activation of downstream ARE-regulated genes (e.g., HO-1, NQO1). Therefore, Nrf2/ARE activation should be considered a suggested or potential pathway rather than a confirmed mechanism. Similarly, although the observed changes in Bax and Bcl-2 expression are consistent with mitochondria-mediated apoptosis, additional markers such as cleaved caspase-3 or cytochrome c would be required to confirm this pathway (7, 24). Our Western blot findings are consistent with Nrf2 upregulation by BSFL extract, supporting this proposed mechanism.

Histopathological examination further supported the biochemical findings. ACR induced severe testicular damage, including disorganized seminiferous tubules, degeneration of the germinal epithelium, and inflammatory infiltration. Treatment with BSFL extract, particularly at the higher dose, markedly improved tissue architecture by restoring tubular organization, epithelial height, and sperm content. These findings are consistent with the protective effects of lauric acid and other bioactives on testicular morphology in models of reproductive injury (14, 15). This morphological recovery underscores the therapeutic potential of BSFL extract for managing ACR-induced testicular damage.

The broad-spectrum protection conferred by BSFL extract is likely related to its distinctive bioactive composition. The n-hexane extract is rich in medium-chain fatty acids, particularly lauric acid (48%), along with oleic acid (12%), palmitic acid (16%), and myristic acid (9.9%). Lauric acid, the predominant component, is well documented for its antioxidant, anti-inflammatory, and anti-apoptotic properties (13, 15-17). Nevertheless, because this study evaluated a complex extract rather than purified lauric acid, the contribution of other bioactive constituents cannot be excluded. The protective effects likely result from synergistic interactions among multiple lipid components, including other fatty acids and minor constituents such as tocopherols and phospholipids (12, 14). Such interactions may underlie the robust protective outcomes observed.

The dose-dependent nature of the protective response is notable. The lower dose (180 mg/kg) provided partial protection, whereas the higher dose (360 mg/kg) was substantially more effective, consistent with previous studies of lauric acid and natural extracts (14, 15). Importantly, BSFL extract alone did not affect any measured parameters, confirming its safety and tolerability.

Our study has several strengths. First, the BSFL extract was extensively characterized, with a comprehensive fatty acid profile confirming its high content of bioactive lipids. Second, we assessed a wide range of hormonal, oxidative, inflammatory, apoptotic, and histopathological markers, providing a holistic view of ACR-induced testicular toxicity and the protective effects of BSFL extract. Third, the dose-dependent protection adds robustness to our conclusions.

### 5.1. Study Limitations

Nevertheless, several limitations should be acknowledged. First, the specific active compounds responsible for the protective effects were not isolated; although lauric acid is likely a key contributor, other bioactive constituents may also contribute, highlighting the need for future bioassay-guided fractionation studies. Second, our investigation focused on the Nrf2 and Bax/Bcl-2 pathways; however, other signaling cascades, such as PI3K/Akt and MAPK, may also be involved and warrant further investigation. Third, the study was conducted in a rodent model with a relatively small sample size (n = 7 per group); additional studies in higher animal models or clinical trials are required to confirm translational potential. Fourth, although our biochemical and histopathological findings indicate promising protective effects, we did not directly assess reproductive functional outcomes such as sperm count, sperm motility, sperm morphology, or fertility. These functional endpoints would be valuable in future studies to determine whether the observed biochemical and histological improvements translate into meaningful improvements in male reproductive function (6-8). Fifth, the use of a single animal species (Wistar rats) limits the generalizability of our findings; future studies using other animal models or strains would be useful to confirm the consistency of the protective effects.

### 5.2. Conclusions

This study demonstrates that the n-hexane extract of black soldier fly larvae (*Hermetia illucens*) exerts significant protective effects against ACR-induced testicular toxicity in male rats. Our findings indicate that BSFL extract, particularly at the higher dose of 360 mg/kg, effectively ameliorated ACR-induced hormonal imbalances by restoring serum levels of FSH, LH, and DHT. In addition, the extract mitigated oxidative stress by reducing lipid peroxidation (MDA) and restoring antioxidant enzyme activities (SOD, CAT, GPx) and glutathione (GSH) levels. The anti-inflammatory potential of BSFL extract was supported by the suppression of pro-inflammatory cytokines (TNF-α, IL-6, and IL-1β). Moreover, the extract modulated apoptosis-regulatory proteins by upregulating Nrf2 and Bcl-2 while downregulating Bax, thereby normalizing the Bax/Bcl-2 ratio and protecting testicular cells from apoptosis. These biochemical improvements were corroborated by histopathological findings showing substantial restoration of testicular architecture, including increased germinal epithelial height and improved seminiferous tubule organization.

The protective effects of BSFL extract are likely attributable to its rich bioactive composition, particularly its high lauric acid content (48% of total fatty acids), along with other medium-chain fatty acids and antioxidant compounds. The multitarget mechanisms involving Nrf2 upregulation, NF-κB suppression, and modulation of the Bax/Bcl-2 pathway highlight the therapeutic potential of this sustainable natural product.

Overall, BSFL n-hexane extract, as a well-characterized and cost-effective natural source of bioactive lipids, shows promise as a protective agent against chemically induced testicular damage. Its safety profile, as indicated by the absence of adverse effects in the extract-only group, further supports its potential for future therapeutic applications. However, further studies are required to identify the specific bioactive compounds responsible for the observed effects and to elucidate the precise molecular mechanisms involved.

## Data Availability

The dataset presented in the study is available on request from the corresponding author during submission or after publication.
